# Differential Axial Requirements for *Lunatic Fringe* and *Hes7* Transcription during Mouse Somitogenesis

**DOI:** 10.1371/journal.pone.0007996

**Published:** 2009-11-24

**Authors:** Michael Stauber, Chetana Sachidanandan, Christina Morgenstern, David Ish-Horowicz

**Affiliations:** Developmental Genetics Laboratory, Cancer Research UK, London Research Institute, London, United Kingdom; Texas A&M University, United States of America

## Abstract

Vertebrate segmentation is regulated by the “segmentation clock”, which drives cyclic expression of several genes in the caudal presomitic mesoderm (PSM). One such gene is *Lunatic fringe* (*Lfng*), which encodes a modifier of Notch signalling, and which is also expressed in a stripe at the cranial end of the PSM, adjacent to the newly forming somite border. We have investigated the functional requirements for these modes of *Lfng* expression during somitogenesis by generating mice in which *Lfng* is expressed in the cranial stripe but strongly reduced in the caudal PSM, and find that requirements for *Lfng* activity alter during axial growth. Formation of cervical, thoracic and lumbar somites/vertebrae, but not sacral and adjacent tail somites/vertebrae, depends on caudal, cyclic *Lfng* expression. Indeed, the sacral region segments normally in the complete absence of *Lfng* and shows a reduced requirement for another oscillating gene, *Hes7*, indicating that the architecture of the clock alters as segmentation progresses. We present evidence that *Lfng* controls dorsal-ventral axis specification in the tail, and also suggest that *Lfng* controls the expression or activity of a long-range signal that regulates axial extension.

## Introduction

Somites are repeated epithelial blocks of tissue that differentiate into the segmental units of the axial skeleton (vertebrae, intervertebral discs, ribs), attached skeletal and limb muscles, and additional mesodermal tissues. In the mouse, somites form between embryonic days E7.75 and E13.5 from the unsegmented, mesenchymal presomitic mesoderm (PSM), which lies towards the caudal end of the embryo. During this process, formation of new epithelial boundaries (every 2 h in the mouse) at the cranial end of the PSM generates a new, bilateral pair of somites. Cell migration and proliferation replenish the caudal PSM as the embryo grows [1], [2], [reviewed in 3], [4].

The sequential process of somitogenesis is controlled by a molecular oscillator, the so-called “segmentation clock”, that is characterised by oscillatory transcription of genes in the PSM with the same periodicity as that of somitogenesis [5], [reviewed in 6]. Expression of cycling genes is synchronised between neighbouring cells, but subject to phase delays along the length of the axis so that a wave of transcription appears to sweep cranially along the PSM, concomitant with the formation of a new pair of somites.

The segmentation clock arises via delayed negative feedback, but details of the clock pacemaker circuitry remain unclear [5], [7], [reviewed in 6], [8]. Amongst several oscillating genes that play essential roles in somitogenesis [reviewed in 9] is *Lunatic fringe* (*Lfng*), which encodes a β1,3-N-acetylglucosaminyl-transferase that acts in the Golgi to modify the extra-cellular domain of the Notch receptor before transport to the cell membrane [10], [11], [12]. Glycosylation by Lfng regulates the sensitivity of Notch receptors towards their ligands and, in the PSM, appears to repress Notch signalling. Thus, inactivating *Lfng* causes constant Notch1 activity (as measured by cleavage of its intracellular domain [13]), and ectopic *Lfng* expression depresses Notch signalling in the chick [14].

**Figure 1:**
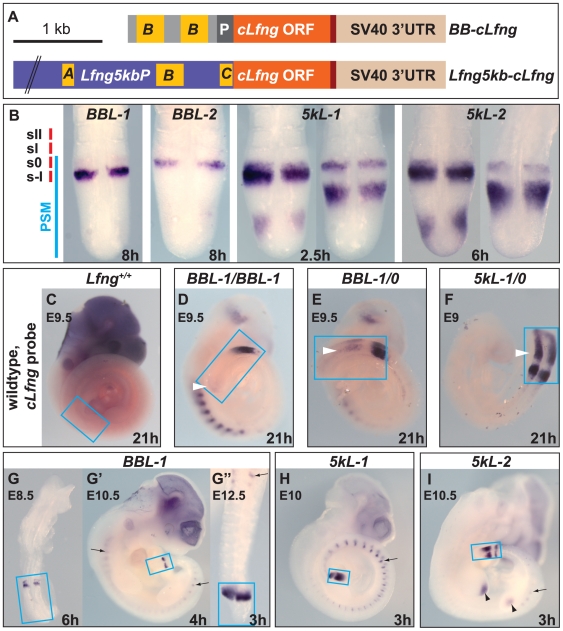
Expressing transgenic *cLfng* in the cranial PSM stripe. (A) Constructs *BB-cLfng* and *Lfng5kb-cLfng* were used to generate transgenic lines *BBL-1/-2* and *5kL-1/-2*, respectively. *cLfng* ORF (orange) is under the control of either a duplication of the stripe-specific B-block enhancer and the *β-globin* basic promoter *P* (top), or 5 kb of the mouse *Lfng* promoter containing conserved cis-regulatory elements A-, B-, C-blocks (bottom; [15]). (B) The resulting transgenic expression (visualised by *in situ* hybridization with a *cLfng* probe on PSMs of hemizygous E10.5 embryos of lines *BBL-1*, *BBL-2*, *5kL-1* and *5kL-2*) is confined to the cranial PSM stripe in *BBL* embryos or comprises stripe plus oscillatory domain in *5kL* embryos. Position of PSM, somitomeres s-I and s0 and somites sI and sII are indicated on the left. Note the different expression levels, as reflected by the different times of colour development (in hours) given at the bottom of each panel. (C–F) Extended time of colour development does not detect endogenous *Lfng* expression in wildtype, but unexpected weak and dynamic *cLfng* expression in the caudal and mid-PSM of *BBL* embryos (white arrowheads). (G–I) Transgenic *cLfng* is expressed in the PSM (boxed in C–I) at all stages of segmentation (e.g. G–G″: hemizygous *BBL-1* embryos at embryonic day E8.5, E10.5 and E12.5) and in ectopic expression domains outside the PSM (e.g. H, I: *5kL-1* and *5kL-2*; black arrows and arrowheads). Arrowed domains are seen in all transgenic lines, indicating that the *Lfng* promoter includes repressive regulatory elements that lie outside the proximal 5 kb region.

*Lfng* transcription in the mouse PSM comprises two dynamic domains, a cycling domain in the caudal PSM, and a cranial PSM stripe adjacent to the boundary that is about to form between somitomeres s–I and s0 (Figure 1B). Promoter analysis has revealed that oscillating *Lfng* transcription in the PSM is driven by an assembly of discrete *cis*-regulatory elements, and that a distinct 285 bp element (B-block [15]; block 3 [16]) drives stripe expression.

The relative significance of the two *Lfng* PSM domains is not clear. *Lfng^−/−^* embryos show irregular and incomplete segmentation of the PSM and perturbed cranial-caudal patterning of somites. The resulting vertebral column is truncated and irregular with incompletely formed and fused vertebrae; the thoracic cage is malformed and several ribs are fused [17], [18]. Thus, the oscillating domain could be a component of the segmentation clock and contribute to periodic gene expression. Indeed, cyclic, spatially patterned *Lfng* transcription is essential for proper somitogenesis: continuous *Lfng* overexpression in the whole mouse PSM, both in wild-type and *Lfng^−/−^* embryos, leads to somite and vertebral column defects [19].

The cranial stripe could also be involved in boundary formation, either maintaining a preformed but morphologically invisible metameric pattern in the PSM, defining cranial-caudal compartments in the forming somite, or regulating the generation of intersomitic boundaries. *Drosophila* Fringe is essential for boundary formation in several imaginal tissues [reviewed in 20], and high levels of vertebrate Lfng in the cranial stripe may contribute to formation of the new somite boundary. Indeed, transplantation experiments in the chick support this view: apposition of *Lfng*-expressing cells adjacent to non-expressing cells in the middle of chick somitomere s-II induces formation of an extra somite boundary [21].

In this study, we dissect individual contributions of the two *Lfng* subdomains by generating transgenic mice that express *Lfng* predominantly in the cranial stripe. We show that oscillatory *Lfng* expression in the caudal PSM is required in the cranial body half, and that activity in the cranial *Lfng* stripe is required in the tail. Both modes of *Lfng* activity are dispensable in the intermediate sacral area. Analysis of a hypomorphic *Hes7* allele indicates that requirements for oscillating *Hes7* expression also vary during somitogenesis, suggesting that the gene network regulating segmentation is subject to region-specific influences along the mouse embryonic length axis.

## Results

### Generation of Transgenic Mice Expressing *Lfng* in the Cranial PSM Stripe

We examined the function of *Lfng* in the PSM by driving *Lfng* expression in the cranial PSM stripe via a tandem duplication of the B-block enhancer of the *Lfng* promoter which drives stripe expression more strongly than a single B-block (*BB-cLfng*; Figure 1A; data not shown; [15], [16]). We expressed chick *Lfng* (referred to as *cLfng* for clarity) so that we could distinguish between transgenic and endogenous *Lfng* expression by *in situ* hybridisation. Although mouse and chick Lfng are 70% identical and 87% similar in aminoacid sequence, indicating that they should be functionally equivalent, the genes share less than 60% identity in DNA sequence, sufficient for a *cLfng* probe to be selective for the chick transcript under stringent hybridisation conditions (Methods). As a control, we used a 5 kb fragment of the mouse *Lfng* promoter to drive *cLfng* in both the stripe and the oscillating PSM domains (*Lfng5kb-cLfng*; Figure 1A; [15], [16]).

We obtained 13 transgenic mouse lines with *BB-cLfng* and 8 lines with *Lfng5kb-cLfng*. All 8 *Lfng5kb-cLfng* lines express *cLfng* in the expected wildtype expression pattern: cyclically in the caudal PSM and stably in a stripe in the cranial compartment of somitomere s–I. 9 of the 13 *BB-cLfng* lines express the cranial stripe. Surprisingly, all 9 lines also weakly express *cLfng* dynamically in the caudal and mid-PSM. This staining indeed represents *cLfng* expression because no cross-reaction with endogenous *Lfng* is seen following equivalent staining of wildtype embryos (Figure 1C). Rather, it appears that the duplicated B-block can drive dynamic expression in the PSM, albeit at very low levels (see Discussion).

For further study, we selected two lines (referred to as *BBL-1* and *BBL-2*) in which caudal expression is very weak and visible only after extended staining, and two control *Lfng5kb-cLfng* lines (referred to as *5kL-1* and *5kL-2*) (Figure 1B, 1D–F). All four lines express the transgenes throughout somitogenesis (Figure 1G–G″, and not shown). The 5 kb fragment drives stronger stripe expression than the duplicated B-block (Figure 1B, 1G–I), indicating that the longer promoter includes additional elements that synergise with and enhance stripe expression from the B-block, and *BBL-1* expresses more highly than *BBL-2*. The former is homozygous viable, allowing us to compare hemi- (*BBL-1/0*) and homozygous (*BBL-1/BBL-1*) animals. Only hemizygous *BBL-2* animals were examined because the insertion is homozygous lethal (genotyping was performed following identification of the transgene insertion site; Methods). Together, the transgene lines form an allelic series of *cLfng* stripe expression (*5kL/0*>*BBL-1/BBL-1*>*BBL-1/0*>*BBL-2/0*; Figure 1B).

### *cLfng* Expression in the Cranial Stripe Rescues Embryonic Tail Growth but Not Adult Viability

The various transgenes were crossed with *Lfng^+/−^* mice and the patterning of *Lfng^−/−^;cLfng^+^* offspring (which we refer to by their transgene names) assayed as foetuses and adults. *Lfng^−/−^* mice usually die before weaning, presumably because the deformed axial trunk skeleton reduces the thoracic space and impairs breathing [17], [18]. In our experiments, only three non-transgenic *Lfng^−/−^* animals survived to adulthood (equivalent to 9% of the expected number of homozygous offspring; Table 1), although a much higher proportion of *Lfng^−/−^* foetuses survived till E18.5 (61%; Table 1).

**Table 1:** E18.5 and adult viabilities of transgenic animals in a *Lfng^−/−^* background^1^.

Line^2^	*5kL-1*	*5kL-2*	*BBL-1*	*BBL-1*	*BBL-2*	all lines
Genotype of interest	*Lfng^−/−^; 5kL-1/0*	*Lfng^−/−^; 5kL-2*	*Lfng^−/−^; BBL-1/BBL-1*	*Lfng^−/−^; BBL-1/0*	*Lfng^−/−^; BBL-2/0*	*Lfng^−/−^* 3
Total number of E18.5 (or *adult*) offspring	87 (*155*)	37 (*116*)	23 (*59*)	49 (*49*)	18 (*129*)	214 (*508*)
Number recovered^4^	18 (*11*)	9 (*11*)	7 (*3*)	6 (*2*)	2 (*2*)	9 (*3*)
Number expected^5^	12.9 (*33.2*)	7.5 (*16.1*)	5.7 (*14.7*)	9.2 (*8.4*)	3.2 (*23.1*)	14.9 (*33.3*)
Relative viability	139% (*33%*)	120% (*68%*)	122% (*20%*)	65% (*24%*)	62% (*9%*)	61% (*9%*)

1 Adult data are in brackets and italicised.

2 For each line, *5kL-1*, *5kL-2*, *BBL-1*, *BBL-2*, we crossed parents of the genotype *Lfng^+/−^;cLfng^+/0^* or *Lfng^−/−^;cLfng^+/0^* or *Lfng^+/−^* or *Lfng^+/−^;cLfng^+/+^* (*BBL-1* only, shown in the 4^th^ column) and genotyped the offspring. We never obtained homozygous foetuses or adults of lines *5kL-1* and *BBL-2* (and we were unable to check for homozygosity of line *5kL-1*). For sites of insertion and genotyping see Methods.

3 Numbers for transgene-free *Lfng^−/−^* in the 7^th^ column are pooled from all crosses of all lines.

4 Numbers recovered of the genotype of interest.

5 The expected numbers of offspring of the relevant genotype were calculated separately for each cross to take account of differing parental genotypes (cf. footnote 2), and then combined.

Expressing *cLfng* in both the stripe and the oscillating PSM domains substantially restores adult viability (33% and 68% for *5kL-1* and *5kL-2*, respectively; Table 1; see Methods S1 for a likely reason of the unexpected high lethality of *5kL* mice), and also enhances foetal recovery (Table 1). Thus, chick Lfng is indeed functional in the mouse and can largely substitute for the endogenous protein. Rescue of adult viability when *cLfng* is expressed solely in the cranial stripe is less efficient, (9–24% in *BBL-1* and *-2*; Table 1).

**Figure 2:**
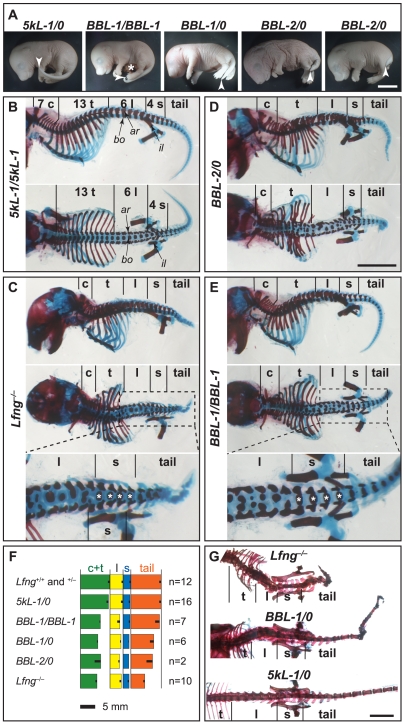
Regional and dose-dependent rescue of the *Lfng* phenotype by transgenic expression in the stripe domain. (A) Dosage-dependent rescue of the truncated tail phenotype of *Lfng^−/−^;cLfng^+^ BBL* E18.5 foetuses. Homozygous *BBL-1* tails resemble those of wildtype/*5kL*, and weak hemizygous *BBL-2* resemble *Lfng^−/−^*. White arrowheads point at the tip of the tails; scale bar, 5 mm; asterisk, midgut that failed to return to the reduced peritoneal cavity (found in 5/42 *Lfng^−/−^;cLfng^+^* E18.5 foetuses). (B, C) E18.5 skeletons of *5kL* embryos show regular vertebrae all along the length axis like wildtype [B, lateral (upper panel) and dorsal (lower panel) view] whereas *Lfng^−/−^* skeletons (C) exhibit regular vertebrae only in the sacral area. (D, E) *BBL* skeletons show a regional rescue in the tail area adjacent to the sacral area. The cervical, *c*; thoracic, *t*; lumbar, *l*; sacral, *s*; and tail regions of the vertebral columns are indicated, as are: *ar*, vertebral arch; *bo*, vertebral body; *il*, ilium. Regular sacral vertebrae in the bottom panels of (C) and (E), magnifications of the middle panels, are labelled with white asterisks. Note the cranial shift of the left side of sacral vertebrae in the *Lfng^−/−^*foetus (C bottom), indicative of homeotic transformation. Scale bar (bottom of D), 5 mm. (F) A schematic quantitative comparison of lengths of cervical/thoracic (*c+t*, green boxes), lumbar (*l*, yellow boxes), sacral (*s*, blue boxes), and tail (orange boxes) sections of vertebral columns of various genotypes indicates a dose-dependent rescue of the tail length in *BBL* foetuses, but not of the length of the cervical+thoracic area. Boxes for each region are aligned to the left (cranial) end. (G) Skeleton preparations of adult mice (dorsal views) show the truncated tail but normal *Lfng^−/−^* sacral area (top), a partial tail rescue in hemizygous *BBL-1* mice (middle) and an overall rescue in *5kL* mice (bottom); scale bar, 10 mm.

The ability of our transgenes to rescue caudal development, as assayed by tail length, correlates with the strength of transgene expression. *Lfng^−/−^* tails are much shorter than wildtype, both at E18.5 and in adult mice (Table 2). This defect is rescued essentially completely by the *5kL* transgenes, and efficiently in mice homozygous for *BBL-1* (Figure 2A and Figure S1A; Table 2). Rescue by hemizygous *BBL-1* and *BBL-2* is intermediate and slight, respectively (Figure 2A; Table 2). Rescued *BBL* tails are often kinked, indicative of locally disrupted somitogenesis due to partially incomplete *Lfng* expression (Figure S1A).

**Table 2:** Comparison of E18.5 foetuses and adults of the four transgenic lines in the *Lfng^−/−^* background (data for adults are given in brackets and italics).

	*Lfng^+/+^* or *Lfng^+/−^*	*5kL-1/0*	*5kL-2*	*BBL-1/BBL-1*	*BBL-1/0*	*BBL-2/0*	*Lfng^−/−^*
Number of E18.5 *(adult)* skeletons analysed	12 (*10*)	16 (*8*)	3 (*7*)	7 (*3*)	6 (*2*)	2 (*0*)	10 (*5*)
Length of whole vertebral column (mm)	27.8±1.4 (*154±5*)	27.1±0.9 (*141±11*)	24.3±4.0 (*148±7*)	22.4±2.4 (*121±3*)	19.8±1.2 (*90±7*)	19.3±4.6	16.0±1.1 (*52±5*)
Length of cervical plus thoracic region (mm)	10.1±0.6	9.7±0.7	8.7±2.1	6.9±0.3	6.0±0.3	7.0±2.1	5.7±0.5
Length of lumbar region (mm)	4.5±0.5	4.4±0.6	4.2±0.3	3.4±0.9	3.8±0.3	3.3±0.4	3.9±0.6
Length of sacral region (mm)	2.7±0.5	2.4±0.4	2.3±0.3	2.4±0.4	2.1±0.2	2.0±0	2.1±0.5
Length of tail (mm)	10.5±0.7 (*92±3*)	10.5±0.7 (*85±9*)	9.2±1.8 (*89±4*)	9.9±1.5 (*74±2*)	7.9±1.3 (*48±4*)	7.5±2.1	4.8±0.5 (*14±3*)
Number of regular sacral vertebrae	4±0 (*4±0*)	4±0 (*4±0*)	4±0 (*4±0*)	4±0 (*4±0*)	3.8±0.4 (*4±0*)	4±0	3.3±0.8 (*3.6±0.5*)
Number of regular tail vertebrae	30.4±0.7 (*28.6±1.5*)	29.6±1.4 (*27.4±3.0*)	18.7±9.1 (*28.4±1.3*)	27.5±1.4 (*23.3±3.1*)	14.5±5.9 (*11.0±1.4*)	6.0±2.8	0.8±1.5 (*0.8±0.4*)
Number of ribs (left and right counted separately)	13±0 (*13±0*)	12.9±0.4 (*12.9±0.9*)	12±1.4 (*13.1±0.5*)	11.1±1.3 (10.6±0.8)	9.6±1.3 (*10.0±1.8*)	10.3±1.5	9.5±1.7 (*10.0±1.3*)

Surprisingly, 15% (3/21) of the *5kL* foetuses show no rescue of tail length (and of skeletal segmentation; not shown). Most likely, these represent individual embryos in which the transgene has been inactivated (see Methods S1), although we cannot exclude the possibility that sequence differences between mouse and chick *Lfng* also contribute to differences between the activity of the transgenic and endogenous genes.

### Stripe Expression of *cLfng* Provides Regionally Restricted Rescue of the *Lfng* Segmentation Phenotype

A single endogenous *Lfng^+^* gene is sufficient for normal skeletal development, irrespective of whether the animals also include a *cLfng* transgene (n = 58). However, *Lfng^−/−^* mice display severely disorganised, truncated axial skeletons with irregular and fused vertebrae and vertebral bodies that rarely align to the midline [17], [18]. They also have an unsegmented tail stump with one or no regular tail vertebrae (Table 2).

Surprisingly, most sacral vertebrae in these animals resemble wildtype [Figure 2C, 2G top; compare sacral (s) to irregular thoracic (t) or lumbar (l) regions] with symmetric bodies and proximal dorsal arches. At least three of the sacral vertebrae form distinct ribs connecting the axial skeleton to the ilium of the pelvic girdle, whereas thoracic ribs and vertebrae undergo multiple fusions in the thorax (Figure 2C, 2G top; Table 2). Relative normality of sacral structures is also evident in previous images of the mutant embryos (e.g. Figure 2B, 2C in [17], Figure 2C in [18]). Thus, the sacral area undergoes considerable segmentation independently of *Lfng* activity.

The *5kL* control transgenes give substantial, albeit sometimes incomplete, rescue of the *Lfng^−/−^* skeleton (Figure 2B, 2G bottom; Table 2). Most *5kL* animals have wildtype-like vertebral columns and the same number of tail segments as wildtype (Table 2), confirming that transgenic *cLfng* is functional in the mouse and showing that the previously described mutant skeletal phenotype is indeed due to disruption of the *Lfng* gene [17].

The rescued mice also frequently exhibit homeotic transformations of single segments: Out of 32 *5kL* mice, 14 (44%) have an extra rib-bearing thoracic vertebra, 15 have an extra pair of “true ribs” (connected to the sternum), and 12 miss a lumbar vertebra. This phenotype is likely to be due to subtle alterations of Notch signalling in the PSM that lead to a delay in *Hox* gene expression in the PSM [22], [23], [24].

Finally, all rescued *5kL* males (*Lfng^−/−^;cLfng^+^*) are sterile (7 *5kL-1*, 10 *5kL-2*), confirming the reported reduced fertility of *Lfng^−/−^* males [25]. This finding excludes the possibility that skeletal malformations contribute to the reduced fertility, and indicates that a testis-specific *Lfng* enhancer lies outside the 5 kb promoter used in the *5kL* lines.

By contrast with the *5kL* transgenes, *BBL* transgenes only rescue segmentation in the tail areas. The sacral area of *BBL* mice is always regularly patterned, as in most *Lfng^−/−^* mice. Cervical, thoracic and lumbar vertebrae are only slightly restored (e.g. dorsal arch quality; Figure 2D, 2E, 2G middle), indicating that oscillating *Lfng* expression is necessary for proper segmentation in these axial regions, and that this requirement cannot be met by the weak dynamic expression in the caudal PSM of these embryos.

However, the stripe domain is sufficient for proper segmentation of the tail [Figure 2D, 2E, 2G middle; compare tail to lumbar area (l) and to the short tail in Figure 2C; Table 2]. The efficiency of tail rescue correlates with the strength of transgene expression (*BBL-1/BBL-1*>*BBL-1/0*>*BBL-2/0*; Figure 2F; Table 2). Even the weakest *BBL-2*-rescued foetus shows three regular tail vertebrae. These results suggest that stripe expression of *Lfng* in the cranial PSM helps maintain somitogenesis in the tail (see below).

### *Lfng* Stripe Expression Restores Somite Organisation in the Tail

The rescue of tail vertebrae by *Lfng* stripe expression could be due to restoration of cranial-caudal compartmentalisation of somites. We tested this idea by visualising expression of *Uncx4.1*, a marker of caudal somite halves [26].

**Figure 3:**
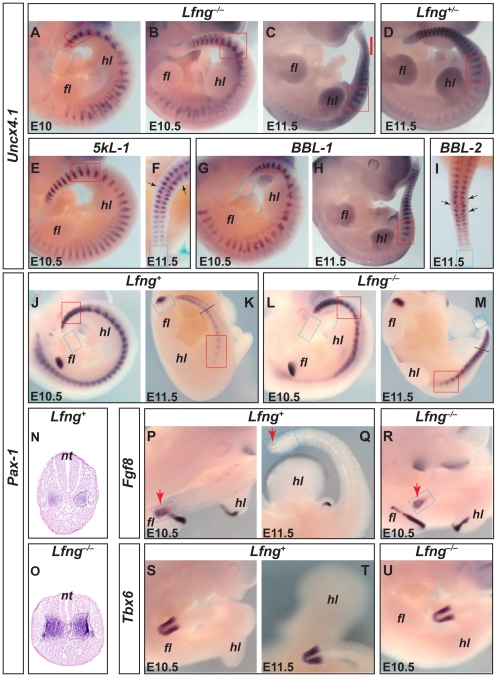
Influence of *Lfng* PSM domains on segmentation and extension of the tail. (A–I) Transgenic *Lfng* stripe expression improves somite compartmentalisation only in the tail. (A–C) Cranial-caudal somite compartmentalisation in *Lfng^−/−^* embryos (visualised by *in situ* hybridization with *Uncx4.1*) is irregular in the cranial body half but regular in the sacral (red box) and first tail somites. *Lfng^−/−^* E11.5 tails are shorter than wildtype-like *Lfng^+/−^* tails (compare C and D). The lack of *Uncx4.1* stripes close to the PSM suggests that segmentation has ceased in the mutant (red bar in C). (E, F) Transgenic *cLfng* expression in *5kL* lines (*5kL-1/0*) almost fully rescues compartmentalisation in trunk and tail (arrows in F indicate occasional irregular stripes). (G–I) Transgenic *cLfng* expression in *BBL* lines (G, *BBL-1/BBL-1*; H, *BBL-1/0*; I, *BBL-2/0*) rescues cranial-caudal compartmentalisation mainly in the tail (arrows in I indicate irregular stripes). (J–O) *Lfng* is important for clear separation of sclerotomes (ventral compartments of somites) except for those in the sacrum, and influences dorsal-ventral patterning of the paraxial mesoderm of the tail. Sclerotomes (stained with *Pax-1*) are regular and segmented in wildtype mice (J, K) but enlarged and undefined in *Lfng^−/−^* embryos except in the sacral area (L, M). 10 µm transverse sections of embryos in K and M at tail region indicated by a dash (N, O; same magnification) counterstained with nuclear fast red (Vector Labs) show an increase in sclerotome size in the mutant (*Pax1*-positive area encircled with a dashed line on right side of sections). (P–U) Expression of PSM markers *Fgf8* (P–R; red arrows point at PSM expression in the tail; expression in Q is weak and restricted, and undetectable in slightly older embryos, not shown) and *Tbx6* (S–U) is indistinguishable between *Lfng^+/−^* and *Lfng^−/−^* embryos. The PSM is boxed in blue. Forelimb buds (*fl*) align to somites ∼8–14, hindlimb buds (*hl*) to somites ∼25–30; *nt*, neural tube.

Segmentation and cranial-caudal differentiation are both greatly disrupted in *Lfng^−/−^* embryos, in which *Uncx4.1* is expressed in irregularly spaced and sized stripes and patches [17], [18]. Nevertheless, from about somite 28 onwards, *Lfng^−/−^* embryos form 17±2 (n = 5) somites that are regular in the sacral and adjacent tail area but become increasingly irregular towards the end of the truncated tail (Figure 3A–3C). The *Uncx4.1* stripes in these somites look slightly less distinct than in wildtype, but are much more regular than in somites 1–27 of the same *Lfng^−/−^* embryos (n = 9; Figure 3A, B).

The cranial somite marker *Tbx18* [27] also reveals relatively regular segments in the sacral and tail area of *Lfng^−/−^* embryos (Figure S2A–D), while the myogenic marker *MyoD* [28] suggests that the regionalisation is less distinct for the myotomal than for the sclerotomal compartment (Figure S2E–H).

Regular segmentation of the sacrum and adjacent tail in *Lfng^−/−^* embryos is not due to compensatory expression of another *fringe* paralogue. Neither *Manic fringe* (*Mfng*) or *Radical fringe* (*Rfng*) is expressed in wildtype PSMs between E8.0 and E11.5 [29, our own observation], and *Mfng* and *Rfng* mRNA levels are not increased in *Lfng^−/−^* embryos compared to wildtype embryos as determined by quantitative RT-PCR on dissected E10.25 PSMs (data not shown).

These relatively well-patterned somites are incorporated into both regular and the severely disorganised *Lfng^−/−^* sacral/tail regions (Figure 2C, 2G top). The latter observation shows that cranial-caudal compartmentalisation is not sufficient for proper development of the sclerotome, which derives from a ventral portion of the somite [reviewed in 3].

*5kL* embryos stain almost normally for *Uncx4.1* (n = 7; Figure 3E, 3F). *Lfng* stripe expression in either *BBL* line also restores orderly *Uncx4.1* expression in the tail (n = 10; Figure 3G–I), albeit less regularly in *BBL-2* tails (Figure 3I), but not in more cranial regions (Figure 3G). These results argue that *Lfng* stripe expression is necessary for tail segmentation.

### *Lfng* Regulates Dorsal-Ventral Compartmentalisation in Tail Somites

As *Lfng^−/−^* tail vertebrae were more irregular than expected from their regular cranial-caudal compartmentalisation (compare Figure 3B and 3C with 2C), we examined sclerotome development in *Lfng^−/−^* embryos using the marker *Pax1* [30], [31]. We found that lack of *Lfng* affects dorsal-ventral compartmentalisation during tail development.

In wildtype embryos, sclerotomes are regular and clearly separated all along the length axis (n = 17; Figure 3J, 3K). In *Lfng^−/−^* embryos, sclerotomes cranial to the hindlimb buds are fused, while the more caudal ones appear distinctly separated (n = 10; Figure 3L, 3M). Sclerotome borders in the mutant never become as clearly visible as in wildtype because *Pax1* expression is less concentrated at the borders, but a relative reduction of *Pax1* in the cranial area is discernible in some sacral sclerotomes (Figure 3L). The *Pax1* domain in the truncated *Lfng^−/−^* E11.5 tails is much thicker than in wildtype tails (compare Figures 3K and 3M and corresponding transverse sections in Figures 3N and 3O), indicating that *Lfng* limits formation of sclerotome. Presumably, over-production of *Pax1*-expressing cells leads to blurring of the borders between sclerotomes in *Lfng^−/−^* embryos (Figure 3L, 3M). This morphological difference between sacral and tail sclerotomes (which is not prefigured by a difference in cranial-caudal compartmentalisation as visualised by *Uncx4.1*; compare Figure 3B, 3C to 3L, 3M) is likely to cause the difference between normal sacral vertebrae and the deformed/fused tail vertebrae in *Lfng^−/−^* mice.

### *Lfng* and Axial Extension of the Tail

*Lfng* plays a role in maintaining axial extension, a process whereby axial progenitor cells which lie at the chordoneural hinge of the tailbud generate caudal growth of the embryo and elongation of the body axis [32], [33], [34]. In *Lfng^−/−^* embryos, axial extension ceases around E10.5, resulting in truncated tails. This phenotype can be rescued by increased *Lfng* expression in the cranial PSM (cf. tail-lengths in hemi- and homozygous *BBL-1* and in *BBL-2* embryos; Figure 2A, 2F; Table 2), indicating that *Lfng* expression in the cranial stripe domain is sufficient for sustained segmentation of the tail. Such a dose-dependent rescue of the length of the vertebral column is not observed in the cranial body half, which is similar in *BBL-1/BBL-1*, *BBL-1/0*, *BBL-2/0* and *Lfng^−/−^* (Figure 2F).

How might cranial *Lfng* expression sustain progenitors in the tailbud? Lfng regulates Notch signalling cell-autonomously [reviewed in 35], suggesting that Lfng activity acts indirectly to influence the behaviour of axial progenitor cells. For example, Lfng activity might be needed to maintain the Fgf and Wnt signalling that is required for axial extension.

To test this idea, we analysed PSMs of *Lfng^−/−^* embryos. Lacking known markers for axial progenitor cells, we analysed the expression of *Fgf8*, a target of Wnt signalling [36], which forms a caudal-cranial concentration gradient and reflects the maturation of the PSM and of the general PSM marker *Tbx6* [37], [38]. *Fgf8* (n = 6) and *Tbx6* (n = 3) expression in *Lfng^−/−^* embryos is indistinguishable from that in control *Lfng^+/−^* embryos (Figure 3P–U) indicating that despite of cessation of progenitor proliferation the *Lfng^−/−^* PSM is still largely intact when segmentation terminates. Thus, *Lfng* is not required to maintain longrange signals of Fgf8, Wnt and retinoic acid in the caudal PSM (see Discussion). We conclude that continued tail segmentation promoted by the cranial *Lfng* stripe in *BBL* mice does not depend on known long-range signals in the PSM.

Further evidence that loss of *Fgf8* expression does not cause the short-tail phenotype comes from our finding that *Fgf8* becomes downregulated between E10.5 and E11.5 in *Lfng^+/−^* embryos (n = 21; Figure 3Q), i.e. about two days before the end of wildtype tail segmentation. This result indicates that other factors control progenitor maintenance from about E11.

### Regular Formation of the Sacral Axial Skeleton Does Not Require *Hes7* Oscillation

The dispensability of *Lfng* for the formation of sacral somites and vertebrae might be due to changing requirements for Notch signalling during axial elongation. To test this idea, we analysed mutants of *Hes7*, a cycling Notch target gene required for somitogenesis that encodes a Notch effector [39], [40], [41]. *Hes7* oscillates in the caudal PSM in phase with *Lfng*, but is not expressed in the cranial PSM [39], [42]. *Lfng* and *Hes7* oscillations depend on each other's expression, consistent with a regulatory loop in which Hes7 protein represses transcription of *Hes7* and *Lfng* and, in turn, Lfng modulates Notch signalling to regulate *Hes7* expression [reviewed in 43].

**Figure 4:**
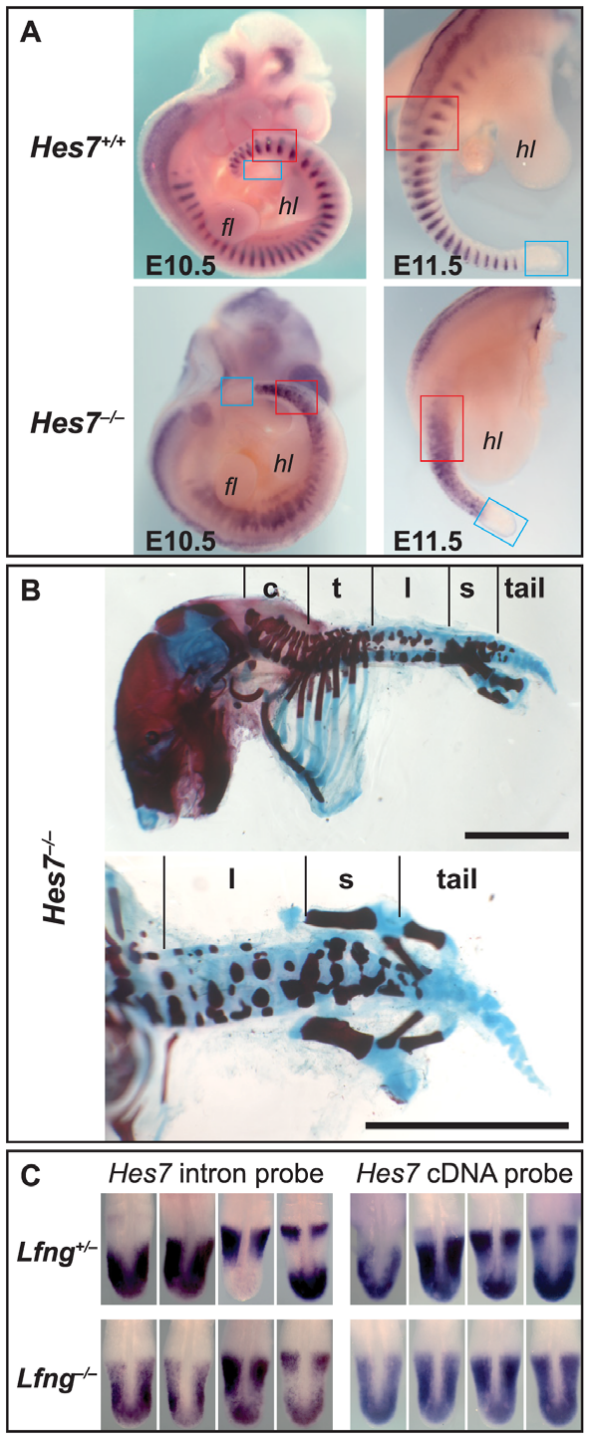
*Hes7* is required for regular sacral segmentation in *Lfng^−/−^* embryos, but need not oscillate. (A, B) Segmentation in *Hes7^−/−^* mice is irregular all along the axis (including the sacrum) as visualised by *Uncx4.1* staining of embryos (A) and foetal skeleton preparation (B; top, lateral view; bottom, ventral view; scale bars are 5 mm). For annotation, see legend to Figures 3 and 2. (C) *In situ* hybridisation with a *Hes7* intron probe to visualise active transcription (left) shows a clearly dynamic pattern in *Lfng^+/−^* PSMs (top) but a strong background throughout *Lfng^−/−^* PSMs (bottom). A *Hes7* cDNA probe (C right) visualises dynamic transcript distribution in *Lfng^+/−^* PSMs (top) but mainly ubiquitous staining in *Lfng^−/−^* PSMs (bottom; embryos showing dynamic expression domain most clearly were selected).

*Hes7^−/−^* E10.5 and E11.5 embryos lack any regular *Uncx4.1* stripes (n = 15; Figure 4A), and *Hes7^−/−^* foetuses do not form regular sacral vertebrae (Figure 4B; [39]). Thus, *Hes7* activity (and presumably its activation by Notch signalling) is required throughout somitogenesis.

Cyclic transcription of *Hes7* is best revealed by *in situ* hybridisation to an intron probe that visualises newly synthesised, unspliced pre-mRNA in the nucleus [15], [40]. Control *Lfng^+/−^* embryos show robust oscillation of unspliced transcripts (and of cDNA; Figure 4C top row). In *Lfng^−/−^* embryos, repression is incomplete so that cyclic *Hes7* expression is superimposed on uniform expression throughout the PSM (n = 49; Figure 4C bottom row, left; Figure S3A,B).

This uniform expression is more evident when probing with an exonic *Hes7* probe. Transcripts are detected throughout the PSM in all *Lfng^−/−^* embryos, and oscillatory transcript accumulation is visible only in few of them (n = 31; Figure 4C bottom row, right; [42], [44]). The largely uniform transcript distribution implies that *Lfng^−/−^* PSMs do not experience dynamic Hes7 protein levels. Presumably, residual dynamic transcription of *Hes7* (as visualised by *in situ* hybridisation with the intron probe) in *Lfng^−/−^* embryos is caused by dynamic transcriptional activation rather than by dynamic transcriptional repression via Hes7 protein. As these mutant embryos form relatively normal sacral segments, *Hes7* expression does not always need to oscillate for proper segmentation. This conclusion is corroborated by our analysis of hypomorphic *Hes7^BAP/BAP^* mice (see below).

### Differential Requirements for *Hes7* Activity During Segmentation in the Sacral Area

**Figure 5:**
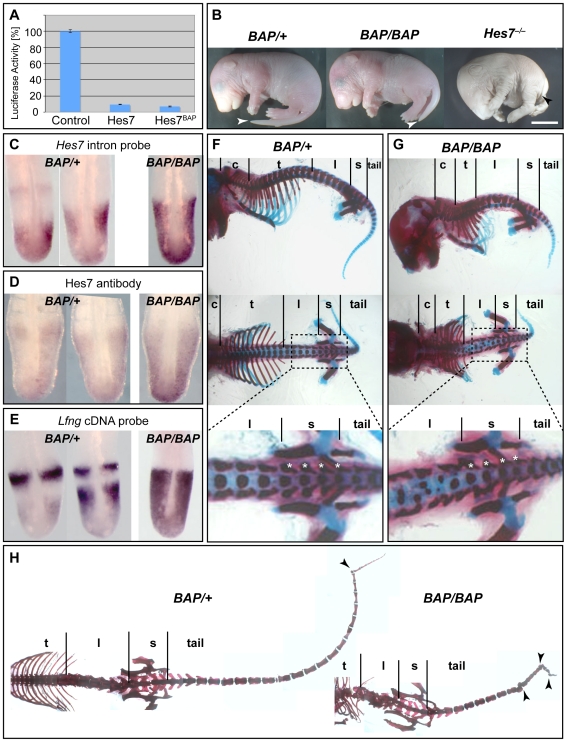
Differential requirement for hypomorphic Hes7^BAP^ during segmentation. (A, B) BAP-tagged Hes7 (Hes7^BAP^) shows full activity in luciferase assays of cultured mouse fibroblast cells (A, relative luciferase activities are shown as mean±s.d. for three experiments) but causes a hypomorphic *Hes7* phenotype *in vivo* (B; note tail lengths of E18.5 foetuses, arrowheads point at the tip of the tails). (C–E) *Hes7* (C, *in situ* hybridisation with an intron probe; D, staining with a Hes7 antibody) and *Lfng* (E, *in situ* hybridisation with a cDNA probe) are oscillating in heterozygous (*BAP/+*; n = 8 *Hes7*, n = 9 *Lfng*) but not in homozygous (*BAP/BAP*; n = 9 *Hes7*, n = 4 *Lfng*) E10.5 embryos. (F–H) Skeletons of heterozygous *BAP/+* foetuses (F) and adults (H left) are regular and resemble wildtype whereas homozygous (*BAP/BAP*) foetuses (G) and adults (H right) are regular only in the sacrum and adjacent tail, similar to *BBL* mice. Foetal skeletons are shown in lateral and dorsal view, adult skeletons in dorsal view; magnification of sacral and adjacent areas of the middle panel in (F,G) are shown in the bottom panel of (F,G); for annotation see Figure 2; arrowheads in (H) point at kinks in tails.

As threshold requirements for *Lfng* activity show axial variations, we hypothesized that the same might be true for *Hes7* activity. Thus, we examined embryos expressing a partial loss-of-function *Hes7* allele in which a 14-aminoacid biotin acceptor peptide (BAP) was knocked into the fourth exon of *Hes7*, 17 aminoacids upstream of the C-terminus. Although the tagged protein (Hes7^BAP^) retains wildtype-like repressor activity in cultured cells (Figure 5A), it shows greatly impaired activity *in vivo*. Only 22% of the expected number of homozygous *Hes7^BAP/BAP^* (*BAP/BAP*) mice survive till weaning (14/254), and the survivors' tails are truncated although not as severely as in *Hes7^−/−^* mice (Figure 5B and Figure S1B, Table 3; [39]).

**Table 3:** Comparison of *Hes7^BAP/+^*, *Hes7^BAP/BAP^*, and *Hes7^KO/KO^* E18.5 foetuses and adults (data for adults are given in brackets and italics).

	*BAP/+*	*BAP/BAP*	*KO/KO*
Number of E18.5 (*adult*) skeletons analysed	3 (*4*)	4 (*5*)	4 (*0*)
Length of whole vertebral column (mm)	30.6±2.3 (*147.5±2.8*)	20±1.9 (*74.4±12.5*)	16.3±0.5
Length of cervical plus thoracic region (mm)	9.3±0.6	5.5±0.6	5.6±0.8
Length of lumbar region (mm)	3.8±0.3	3.6±0.8	4.0±0.4
Length of sacral region (mm)	1.96±0.05	1.6±0.2	1.6±0.3
Length of tail (mm)	11.5±0.5 (*87.25±4.8*)	6.5±2.4 (*32.4±10*)	4.9±0.6
Number of regular lumbar vertebrae	6±0 (*6±0*)	2.3±0.9 (*1±1*)	0
Number of regular sacral vertebrae	4±0 (*4±0*)	3.8±0.5 (*3.2±1*)	0
Number of regular tail vertebrae	28±1 (*27±1.4*)	6.5±4.4 (*4±3.4*)	0
Number of ribs, left and right counted separately	13±0 (*13±0*)	9±0.9 (*9.8±0.8*)	7.3±0.7

*Hes7^BAP^* transcript levels do not oscillate in the *BAP/BAP* knock-in embryos (Figures 5C and S3D,E; n = 27 covering all stages between E9.5 and E11.5). Hes7 protein is expressed uniformly in the whole PSM (Figure 5D, n = 7∼E10.25). Likewise, *Lfng* is expressed continuously throughout the *BAP/BAP* PSM (Figure 5E). A likely explanation for these findings is that BAP-tagging Hes7 has disrupted its repressive activity so that it is unable to maintain an auto-inhibitory feedback loop *in vivo*. This hypothesis is supported by analysis of heterozygous *Hes7^BAP^* (*BAP/+*) embryos, in which *Hes7* and *Lfng* transcript levels are normal and oscillating (Figure 5C, 5E), and by our observation that *Hes7*, a target of its own protein, the repressor Hes7, is upregulated in *BAP/BAP* embryos (Figure 5C,D).

*BAP/BAP* mice display strongly regionalised segmental phenotypes, indicating that *Hes7* activity, like that of *Lfng*, is required differentially during the course of somitogenesis. The mutant mice show severe segmentation defects in the cervical, thoracic and lumbar area with fused vertebrae and fewer ribs (Figure 5F–5H; Table 3). However, the sacral (and to variable degrees, the adjacent lumbar and tail) vertebrae (and *Uncx4.1* stripes; not shown) appear relatively normal, and the tail length is significantly longer than in null mutant mice (cf. Figures 4B and 5F–5H; Table 3). Together, our results indicate that *Hes7* activity is required along the whole body axis for proper segmentation, but that *Hes7* oscillation is not essential during the segmentation of the sacral and adjacent tail area.

## Discussion

By engineering *Lfng* to be expressed in the PSM stripe subdomain with only minimal caudal expression, we have shown that the relative importance of the oscillating and the stripe domains changes as somitogenesis progresses. The cranial half of the axial skeleton appears to rely on the oscillating domain, whereas the tail region needs mainly the stripe domain. Segmentation in an intermediate region requires neither domain. These findings suggest that the molecular circuitry of the segmentation clock changes during the course of development, a view that is also supported by analysis of a *Hes7* hypomorphic allele. We discuss the individual functions of each subdomain during segmentation and tail extension, and the significance of the temporally different segmentation phases.

### Regional Requirements for Different Modes of *Lfng* Expression

An important role for *Lfng* during mouse and chick somitogenesis is indicated by its expression pattern, and by the effects of manipulating its activity [14], [17], [18], [45], [46], [47]. It is less clear if *Lfng* also acts in somitogenesis of lower vertebrates such as zebrafish and medaka, where it is expressed in one or two stripes in the cranial PSM but does not oscillate [48], [49], [50], [51]. Corn snake embryos express *Lfng* in up to nine stripes of variable size and spacing in the mid- and cranial PSM, but they too lack a caudal oscillating domain [52].

Thus, the oscillating, caudal domain may be an evolutionary novelty of higher vertebrates, and the more widely conserved stripe domain may fulfil a more fundamental role during segmentation. Nevertheless, both domains play crucial and distinct roles in mouse segmentation. The oscillating, caudal *Lfng* domain appears essential for the formation of the cranial half of the axial skeleton. Expression of *cLfng* in the cranial stripe fails to rescue somite formation and cranial-caudal patterning of somites 1 to ∼30, and few *BBL* pups, in which this is the only domain of *Lfng* expression, survive to weaning (Figures 2A, 2D–G, 3G–I; Table 1). By contrast, the stripe domain appears to be sufficient and, in the absence of the oscillating domain, necessary for caudal somite formation and patterning, from somite ∼34 onwards (Figures 2A, 2D–G, 3G–I).

Alternatively, the regional phenotypes may reflect different threshold requirements for striped *Lfng* in different body regions. Transgenic *Lfng* expression levels are below wildtype levels, and might be too low for regular segmentation cranial to the sacral somites, but sufficiently high for tail somites. In this scenario, high *Lfng* stripe expression is needed to form somites 1 to ∼30, while weak *Lfng* stripe expression is sufficient for the formation of tail somites from somite ∼35 on. However, requirement of oscillatory *Lfng* for normal segmentation of the cranial body has been demonstrated previously [14], [19].

Shifley *et al.* [53] recently reached similar conclusions to our former model by analysing mice in which *Lfng* PSM-expression is restricted to the stripe domain. They studied mice (*Lfng^ΔFCE1^*) in which FCE1/A block, the major enhancer element driving caudal oscillating expression, has been deleted from the endogenous *Lfng* locus. They too found that *Lfng* stripe expression rescues caudal but not cranial segmentation, indicating that *Lfng* requirements differ between trunk and tail formation.

Although a single B-block drives expression only in a cranial stripe, our transgenic *BBL* lines (with tandem B-blocks) also express very low levels of dynamic–presumably oscillating–*cLfng* in the PSM. Multiple transgenic lines show such expression, indicating that it is indeed due to the duplicated B-block, not the effects of chromosomal flanking sequences on the transgene, i.e. the B-block includes a cryptic oscillator element whose ability to express is enhanced by the tandem duplication. This result is consistent with previous evidence that the *Lfng* promoter includes multiple cyclic enhancers. In particular, *Lfng* reporter transgenes lacking the A block still cycle in the caudal PSM, albeit weakly (*mlf(1.8)*; [15]). Such expression is visible only after extended staining, perhaps explaining why such cyclic expression was not observed in the *ΔFCE1* embryos [53].

This weak cyclic expression is unlikely to contribute to the rescue of tail segmentation in *BBL* and *ΔFCE1* mice. For this to be the case, segmentation of the trunk would have to require more oscillatory *Lfng* than tail segmentation, although the intermediate sacral region does not require oscillatory *Lfng* at all. The oscillatory caudal expression in *BBL* embryos is insufficient to rescue segmentation in the trunk (Figures 1D, 1E, 2C–G) and to maintain *Hes7* oscillation (Figure S3C). Most likely, restoration of the tail is indeed due to the strong, cranial stripe domain of *Lfng* expression.

Unexpectedly, the sacral region segments almost normally in *Lfng^−/−^* embryos: somites ∼31 to ∼34 are regular and cranial-caudal compartmentalised, and form normal vertebrae (Figures 2C, 2G, 3A–C). The ability of this region to segment independently of *Lfng* activity has not been noted previously, and is further evidence that the segmentation circuitry varies along the cranial-caudal body axis. Although Hes7 activity is needed for formation of these segments [39], such activity needs not cycle: *Hes7* cycling is lost in both *Lfng^−/−^* and *Hes7^BAP/BAP^* embryos (Figures 4C, 5C, 5D, S3B, S3E), yet sacral segments form normally.

Requirements for Hes7 cycling protein show a similar axial variation. Segmentation in *Hes7^BAP/BAP^* embryos is rescued only around the sacral and adjacent tail region (Figure 5G, 5H right), indicating that this region requires less Hes7 activity than elsewhere.

Two lines of evidence argue that Hes7^BAP^, in which a peptide tag is inserted near the C-terminus, is almost completely inactive *in vivo*, even though it retains repressive activity in cultured cells. The homozygous *Hes7^BAP/BAP^* phenotype is almost as strong as that of *Hes7^−/−^* embryos, and is fully recessive. *Hes7* is overexpressed in homozygous but not heterozygous *BAP* embryos, indicative of a recessive failure of repression.

The apparent difference between the *ex* and *in vivo* activity of Hes7^BAP^ could be due to the considerable overexpression in the former context that might allow a greatly weakened Hes7^BAP^ still to repress target genes. Alternatively, the insertion in Hes7^BAP^ prevents binding of an accessory factor that is only required in the authentic, *in vivo* environment. Clearly, because a protein is active in a cultured cell assay, it cannot be assumed to retain activity *in vivo*.

### Temporal Changes of the Segmentation Machinery

Axial changes in the segmentation circuitry were first suggested by the phenotype of Notch signalling mutants. The first 7–9 somites form normally in *Notch1*/*Notch2* double knock-out mutants and in mice lacking the common, downstream transcription factor RBP-Jk/CBF1 [54], [55]. Notch pathway mutations in zebrafish also retain the first ∼7 somites [56], [57]. However, this apparently regional phenotype might be due to gradual desynchronisation of neighbouring oscillators during axial elongation, not to changes in regulatory circuitry [58]. An example of differential regulation of mesoderm development along the length axis is the changing hierarchy of the T-box genes *no tail*, *spadetail* and *tbx6* in trunk versus tail in zebrafish embryos [59]. Several other genes exert regionalised effects on somite formation by unclear mechanisms, e.g. *α5-integrin* [60].

Our results define at least three phases of segmentation during the 5 days of mouse somitogenesis (Figure S4). Phases A (somites 1-∼30; E7.75–E10) and C (somites ∼35–65; E10.5–E13.5) rely mainly on oscillating *Lfng* and the cranial PSM stripe, respectively, and have also been described by Shifley *et al* [53]. However, we also see clear evidence of a transition phase B (somites ∼31-∼34) that is associated with a drop in the requirement for *Lfng* activity such that, even in the complete absence of Lfng, mice frequently develop normal sacral vertebrae. This sacral region is also less sensitive to the reduced levels of Hes7 activity in homozygous *Hes7^BAP^* animals (Figure 5G, 5H right). *Hes7* requirements may be more stringent for vertebrae in the tail; 43% of adult *Hes7^+/−^* animals have a kinked tail [39].

As our *BBL* mice express some cyclic *Lfng* in addition to the cranial PSM stripe that is probably below wildtype levels, we cannot formally exclude the alternative but unlikely model (see discussion above) that stronger, wildtype-like stripe expression would alleviate the *Lfng* phenotype in the trunk, or that the weak oscillating caudal domain in *BBL* embryos contributes to the tail rescue. A transgenic line expressing only the cranial PSM stripe at wildtype levels without a caudal PSM domain would allow to unambiguously assign individual expression domains (oscillatory vs. stripe) to phases A and C. However, such a desirable transgenic line cannot be made because the *Lfng* promoter is more complex than previously thought: the “stripe-specific” enhancer (B-block) also drives a faint caudal domain, and other promoters are unlikely to precisely match timing, position, extent and expression level of the wildtype *Lfng* stripe.

Requirements for Wnt signalling, which is needed for mouse *Lfng* to oscillate [61], [62], might also show regional variation. Mice with reduced canonical Wnt signalling due to an in-frame insertion of *lacZ* in the *Lef1* gene form skeletons with severely disorganised vertebrae. These mice exhibit a regularly patterned sacral and cranial tail region similar to that found in *BBL* animals (Figure 1B in [63]).

It is unclear what positions and triggers the different phases. In wildtype mice, phase B is inconspicuous, and not revealed by any obvious variability in oscillatory modes of expression. The beginning of phase B coincides with the time when mesodermal cells are recruited from the tailbud and no longer by involution and streaming through the primitive streak as for phase A (∼30-somite stage; [64]). The phase B-mode could lead to a partial loss of synchronisation of PSM cells due to changed cell movements, and the segmentation clock circuitry might be modified to take this into account.

The onset of phase B is slightly variable: the site of the first regular vertebra in *Lfng^−/−^* mice varies between the last two lumbar vertebrae and the first two sacral vertebrae. Thus, the lumbosacral boundary, which is positioned by the activity of *Hox9* and *Hox10* paralogous genes in the PSM [22], [24], does not directly determine the onset of phase B. Nevertheless, the number of vertebrae covered by phase B is relatively invariant (4.1±1.6; details in Table 2), indicating that the duration of the phase is regulated either via the segmentation clock, or by another process independent of axial patterning, e.g. morphological changes associated with the end of gastrulation.

### *Lfng* Is Important for the Maintenance of Somitogenesis

Premature termination of axial extension in *Lfng^−/−^* embryos indicates that progenitor cells become depleted in these embryos at around E10.5. The low variability of *Lfng^−/−^* tail lengths hints at a clear-cut temporal change in the requirement of *Lfng* for maintenance of axial extension in trunk vs. tail when the segmentation machinery shifts from phase B to C.

Our transgenic *BBL* lines show an unexpected *Lfng* expression domain in the caudal PSM. Although this caudal domain could contribute to continued axial extension of the tail by short-range signalling, its expression is very weak and unable to promote segmentation (discussed above). Rather, the rescue of the *Lfng^−/−^* short-tail phenotype by stripe expression of *Lfng* in *BBL* mice suggests that *Lfng* expression in the cranial PSM regulates expression of a component that signals caudally to regulate axial extension.

Our results suggest that this signal is not mediated by retinoic acid, a long-range signal made in newly formed somites; retinoic acid regulates expression of *Fgf8* in the caudal PSM and tailbud [65], [66], yet *Fgf8* expression is normal in *Lfng^−/−^* E10.5 embryos (Figure 3P, 3R). Either retinoic acid signalling is unaffected in the mutant embryos, or Lfng modifies retinoic acid expression or activity in a manner that does not affect *Fgf8* transcription.

Indeed, Fgf8 transcription may not be required to sustain proliferation of axial progenitor cells at late stages of somitogenesis. *Fgf8* transcripts are no longer detectable in wildtype PSMs from stage E11.5 (Figure 3Q), yet somitogenesis and *Tbx6* and *Lfng* transcription persists for a further two days (Figure 3T, and not shown). This lack of linkage between Fgf and *Lfng* expression suggests that other signals from the cranial PSM or somites are involved in maintaining axial elongation.

## Materials and Methods

### Mouse Lines

The *Lfng* null line was obtained from Randy Johnson [17] and the *Hes7* null line from Ryoichiro Kageyama [39]. Transgene constructs were injected into *F1×F1 (CBA×C57Bl/6J)* mouse embryos. Transgenic mice were kept in the *C57Bl/6J* background. *C57Bl/6J* also served as wildtype control. All animals were handled in strict accordance with good animal practice as defined by the Animals (Scientific Procedures) Act 1986 and in accordance with the Codes of Practice issued for the use of animals in scientific procedures issued by the UK Home Office. All animal work was approved by the ethical committees for experiments with animals of the London Research Institute.

### Preparation of *cLfng* Transgene Vectors and *Hes7^BAP^* Targeting Construct

The construction of transgene vectors *BB-cLfng* and *Lfng5kb-cLfng* (Figure 1A) and of the targeting vector used to generate *Hes7^BAP^* mice is described in Methods S1.

### Thermal Asymmetric Interlaced (TAIL) PCR

We mapped the integration sites in the transgenic lines by amplifying the flanking genomic regions using TAIL-PCR with a combination of degenerate and nested primers ([67]; for primers see Table S1).

In *5kL-1* mice, the transgene is inserted into chromosome 19, position 31,973,131 (Build 36), i.e. into the second intron of the *APOBEC-1 complementation factor* (*ACF*) locus. We never obtained homozygous transgenics from various hemizygous *5kL-1* parents (100 offspring genotyped for homozygosity), consistent with the pre-implantation lethality of homozygous *ACF^−/−^* embryos [68].

The *BBL-1* transgene is inserted into chromosome 8, position 44,990,545. Homozygous *BBL-1* transgenics are viable and fertile and appear normal.

In *BBL-2* we found three integration sites located within a distance of 500 kb on chromosome 4, all in introns of the “unclassifiable” cDNA *AK139518* (positions 23,086,138/23,085,307; 22,619,012; 22,600,785). Integration in position 23,085,307/23,086,135 resulted in a rearrangement of the genomic locus. No homozygous transgenic mice of *BBL-2* were identified among the offspring of several hemizygous parents (46 offspring genotyped for homozygosity), indicating that homozygous insertion is embryonic lethal.

We were unable to identify the genomic integration site of the *5kL*-2 line, but found tandem insertions of the transgene, as were also present in the other three lines.

### *In Situ* Hybridisation and Immunochemistry

Whole-mount *in situ* hybridisation was performed by a modification of the method of Henrique *et al.* ([69]; described in the Supporting Information). RNA probes were made from 2.1 kb *cLfng* cDNA [70], 0.7 kb *Fgf8* cDNA [71], 0.7 kb *Hes7* ORF [72], 1.2 kb mouse *Lfng* cDNA (IMAGE clone 408467), 1.9 kb *MyoD* cDNA [28], 0.9 kb *Pax1* cDNA [30], 0.7 kb *Tbx6* cDNA [38], 1.4 kb *Tbx18* (PCR-amplified from exon 8) [27], and 0.7 kb *Uncx4.1* cDNA [73]. The *Hes7* intron probe was synthesised from a 1 kb PCR product of the first intron cloned into *pCRII-TOPO* vector (Invitrogen) and used as for the cDNA probes except that hybridisation was at 65°C instead of 70°C. Hes7 antibody staining was performed as described [40].

### Skeleton Preparation

Adult and E18.5 skeletons were prepared and stained with alcian blue/alizarin red S following standard procedures. All photos were taken with a Leica DC500 digital camera and Leica Firecam version 1.7.1 software. Several photos were assembled for adult skeletons in Figure 2G and 5H.

### Luciferase Reporter Assay

C3H10T1/2 cells were plated at a density of 8×10^4^ per well of a 24-well tissue culture plate 24 hours before transfection. 100 ng of the firefly luciferase reporter under the control of six N-boxes and the *β-actin* promoter [74] was cotransfected with 200 ng *pCI* (Promega), *pCI-Hes7* or *pCI-Hes7-BAP* using GeneJuice (Novagen) transfection reagent at a ratio of 1∶3, DNA∶GeneJuice. 4 ng of the *Renilla* luciferase vector *pRL-TK* (Promega) was used in each sample as reference reading. After incubation for 24 hours at 37°C and 5% CO_2_, the assay was analysed using the Dual-Luciferase Reporter Assay System (Promega) according to the manufacturer's guidelines. *Firefly* and *Renilla* activities were read using the EnVision Multilabel Reader. The values of the reporter readings were normalised to the values of the *Renilla* reading. The resulting luciferase activity alone was taken 100%. Each experiment was done in triplicates and repeated at least three times.

## Supporting Information

Figure S1 Adult tail phenotypes of 5 kL and BBL transgenes and Hes7BAP knock in mice. Transgenic mice of the 5 kL lines (without endogenous Lfng) resemble wildtype and Lfng+/−mice; BBL lines (without endogenous Lfng) show a tail rescue of variable degree (A, arrowheads point at kinks). Hes7BAP/+ mice resemble wildtype; Hes7BAP/BAP mice have truncated, kinky tails (B).
(3.34 MB TIF)

Figure S2 Expression of Tbx18 and MyoD in Lfng−/− E11.5 embryos. Expression of Tbx18 (A–D) and MyoD (E–H) in Lfng+ and Lfng−/− E11.5 embryos as indicated. Tbx18 is expressed in regular stripes along the length axis of wildtype embryos (A, B); Tbx18 stripes in mature somites are broad but separate (e.g. red arrow). Tbx18 domains in the trunk of Lfng−/− embryos (C, D; n = 4) are fused and irregular (e.g. region labelled by red bar). More distinct Tbx18 domains are visible in the sacral (boxed) and tail (e.g. black arrows) region of these embryos, resembling Uncx4.1 expression (cf. Figure 3C); hl, hindlimb bud. (E–H) MyoD stripes appear generally more regular than Uncx4.1 stripes in Lfng−/− E11.5 embryos (G, H; n = 3; compare to Figure 3C), but less stripes are present in Lfng−/− as compared to wildtype (E, F). Regionalisation of the Lfng−/− null phenotype visualised by MyoD is less obvious than in Uncx4.1 or Tbx18 stainings, i.e. fused stripes can be observed in any region of the length axis including the sacrum, indicating that the observed requirements for Lfng activity mainly apply to the sacral, and not to the myotomal compartment.
(4.32 MB EPS)

Figure S3 Non-oscillatory Hes7 expression in E11.5 Lfng−/−, BBL-1, and BAP/BAP embryos. Transcription of Hes7 visualised by in situ hybridisation of Lfng+/− (A), Lfng−/− (B, n = 5), BBL-1 (C, n = 4), BAP/+ (D) and BAP/BAP (E, n = 5) E11.5 embryos with a Hes7 intron probe. Two to three examples are shown for each genotype. Unlike the results reported in [53], we find that Hes7 expression is not dynamic in Lfng−/− (B), BBL-1 (C), and BAP/BAP (E). Black arrows point at regular somite borders in an embryo with uniform Hes7 expression throughout the PSM in (C).
(4.23 MB EPS)

Figure S4 Three phases of mouse segmentation. Segmentation phase A, which comprises the cervical, thoracic and lumbar region, requires oscillatory Lfng and oscillatory Hes7+. During phase B, the segmentation of the sacrum, Lfng expression in both PSM domains is dispensable; Hes7 does not need to have full activity (as in the hypomorphic allele Hes7BAP) and does not need to oscillate. Phase C, segmentation of the tail, requires the cranial Lfng stripe expression and oscillatory Hes7+, but the oscillatory Lfng domain is less important.
(0.45 MB EPS)

Table S1 List of primers.
(0.08 MB DOC)

Methods S1 (0.03 MB DOC)
