# Supplementary figures and images for: Differential Axial Requirements for Lunatic Fringe and Hes7 Transcription during Mouse Somitogenesis
Source: PLoS One. 2009 Nov 24;4(11):e7996. doi: 10.1371/journal.pone.0007996 (PMC2776510; doi:10.1371/journal.pone.0007996)

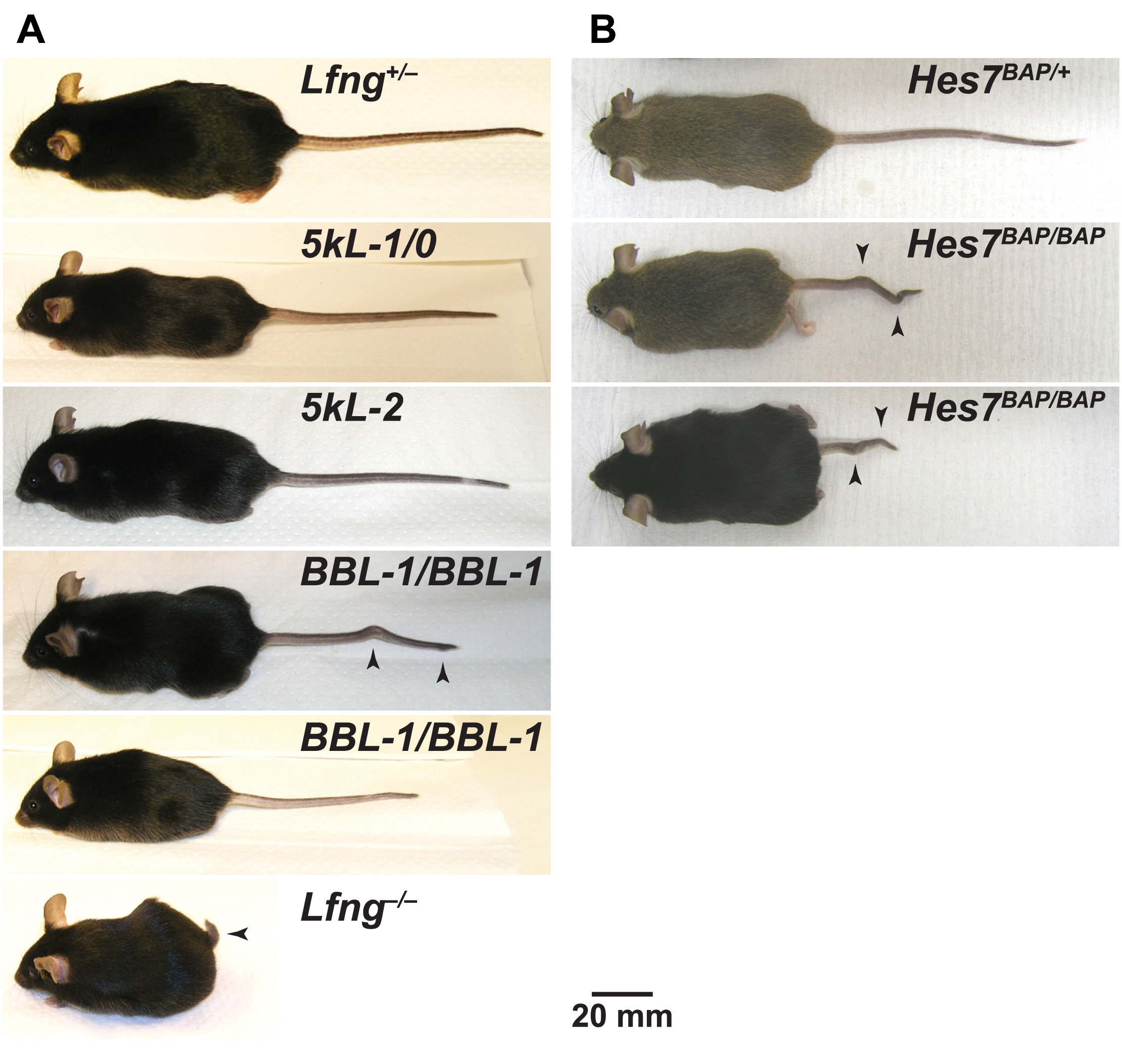

Supplement: Figure S1 — Adult tail phenotypes of 5 kL and BBL transgenes and Hes7BAP knock in mice. Transgenic mice of the 5 kL lines (without endogenous Lfng) resemble wildtype and Lfng+/−mice; BBL lines (without endogenous Lfng) show a tail rescue of variable degree (A, arrowheads point at kinks). Hes7BAP/+ mice resemble wildtype; Hes7BAP/BAP mice have truncated, kinky tails (B). (3.34 MB TIF) [file pone.0007996.s001.tif]
