# Supplementary material for: Differential Axial Requirements for Lunatic Fringe and Hes7 Transcription during Mouse Somitogenesis
Source: PLoS One. 2009 Nov 24;4(11):e7996. doi: 10.1371/journal.pone.0007996 (PMC2776510; doi:10.1371/journal.pone.0007996)
Supplement: Table S1 — List of primers. (0.08 MB DOC) [file pone.0007996.s005.doc]

| **Primer pairs for genotyping** | **forward** | **reverse** |
| --- | --- | --- |
| *Lfng* | TTGGCTTGTCTCCTGGTGCTCACG | AGCAGCCACACGGGGCTGTAATGG |
| *cLfng* | TGACAAGTTCATCGAGTCTGGGAGAAAG | CGTCGAAGATGTTGGGGTGTTGTAAC |
| *neo* (*Lfng* knock-out) | TGGGCACAACAGACAATCGGCTGCTCTG | CGGCAAGCAGGCATCGCCATGGGTC |
| Biotin Acceptor Peptide (BAP) | CCCATCCCACTGCTCCTCTC | AGAAAGCGATTCAAAGGTTGTGG |
| Homozygous BAP | CCCATCCCACTGCTCCTCTC | GGATCCCTCTCCTGCCCTCT |
|  |  |  |
| **Primer pairs flanking genomic insertion sites** | **forward** | **reverse** |
| *BBL-1* (385-387) | ACCCAACCCACCTGTTGTTTACC | CCAATCACAAAAGAACACACATGG |
| *BBL-2* a (388-389) | CCCAGCAGCTACTGTTTCACTCC | CCTCTCTCCTTTCTCAGTCCAAACC |
| *BBL-2* b (413-415) | TCCATTCCTGTGGCCTAGTTGG | CTGCATGCATAAACAAGTCCATAGTC |
| *BBL-2* c (416-418) | TTGGGCTTGTACCCAAAAGACTC | TGCCAAGCTGAAGAACTCACCA |
| *5kL-1* (392-394) | CCAATGTATCACCCTACCCGTCCTC | TGGGCTATAGATGCCACATTCTTGG |
|  |  |  |
| **Arbitrary degenerate TAIL primers** |  |  |
| *AD1* | NTCGASTWTSGWGTT |  |
| *AD2* | NGTCGASWGANAWGAA |  |
| *AD3* | WGTGNAGWANCANAGA |  |
| *AD8* | NTATGWGSTWTSAGC |  |
|  |  |  |
| **Construct-specific TAIL primers** |  |  |
| *5’tail-1* | TTAATGCGCCGCTACAGGGCGCGTC |  |
| *5’tail-2* | AATGTGCGCGGAACCCCTATTTG |  |
| *5’tail-3* | AATATGTATCCGCTCATGAGACAATAAC |  |
| *5’tail-4* | CGACCTACACCGAACTGAGATAC |  |
| *5’tail-5* | TACAGCGTGAGCTATGAGAAAG |  |
| *5’tail-6* | AACGCCTGGTATCTTTATAGTCC |  |
| *3’tail-1* | TTGCTTGCTTTGCTATTTACACCAC |  |
| *3’tail-2* | GAAAAAGCTGCACTGCTATACAAG |  |
| *3’tail-3* | TGTAACCTTTATAAGTAGGCATAAC |  |
|  |  |  |
| **Primer pairs for the *Hes7* targeting construct** | **forward (*EcoR*V site in bold, BAP-tag sequence in italics)** | **reverse (*EcoR*V site in bold, BAP-tag sequence in italics)** |
| Hes7^BAP 5’HR^, (5’ homology region) | CCTGCAGGGAGTGAGAGGGAAACGAATGG | TCTAGAGACACGCGCGGGTGTTATTAACC |
| Hes7^BAP 3’HR upper^, (upper part of 3’ homology region) | CTCGAGTGTCTCTGTGTCTCCCTCATTG | ***GATATC****ATTCAGGCC*CCCGTCTTGTCTGTAAGGCGGTG |
| Hes7^BAP 3’HR lower^, (lower part of 3’ homology region) | ***GATATC****TTTGAGGCCCAGAAGATCGAGTGGCAT*GCGCCCAAGGCCCCGTCACTCC | AAGCTTCCACTGGTAGCAGGGAAAGTGG |
