# Supplementary material for: Differential Axial Requirements for Lunatic Fringe and Hes7 Transcription during Mouse Somitogenesis
Source: PLoS One. 2009 Nov 24;4(11):e7996. doi: 10.1371/journal.pone.0007996 (PMC2776510; doi:10.1371/journal.pone.0007996)
Supplement: Methods S1 — (0.03 MB DOC) [file pone.0007996.s006.doc]

**Supplemental Methods**

**Protocol 1. Preparation of transgene vectors *BB-cLfng* and *Lfng5kb-cLfng*.**

For construction of the *BB::cLfng* plasmid we used the *pβgal-basic* vector (BD Biosciences), a mammalian β-Galactosidase reporter vector without eukaryotic promoter. We inserted a tandem duplication of the *Lfng* stripe-specific enhancer “B-block” (285 bp fragment; [15]). Insertion of a *Hin*dIII-*Xba*I-*Cla*I linker into the *Hin*dIII/*Cla*I sites completely removed the *lacZ* reporter gene, but retained the SV40 3’UTR, resulting in the *BB-Hind*III*Xba*I*Cla*I*-SV40pA* plasmid. A basal *β-globin* promoter was cloned into the *Hin*dIII site and a C-terminally FLAG-tagged chick *Lfng* (*cLfng*) ORF from an RCAS-expression vector (gift from O. E. Orozco and C. Tabin; [70]) inserted into the *Cla*I site. The resulting *BB::cLfng* construct (Figure 1A) was linearised with *Sca*I/*Sal*I, removing a 1.6 kb vector fragment for injection.

For construction of the *Lfng5kb::cLfng* vector we PCR-amplified a 5 kb fragment of the *Lfng* promoter containing the endogenous basal promoter and cloned it into *TOPO-II (2.1)* vector (Invitrogen). The 5 kb promoter fragment was excised with *Sma*I/*Xho*I and cloned into the *Sma*I/*Xho*I sites of the *BB-HindIIIXbaIClaI-SV40pA* plasmid (see above), replacing the duplicated B-block. The C-terminally FLAG-tagged *cLfng* ORF from the RCAS-expression construct was introduced via the *Cla*I site as for the *BB::cLfng* vector. The resulting *Lfng5kb::cLfng* construct (Figure 1A) and linearised with *Sal*I for injection. The vector sequence could not be removed due to a mutation in a restriction site, possibly leading to unreliable transgene expression in *5kL-1* and *5kL-2* mice and their unexpected high lethality despite the observed wildtype-like patterning and gene expression.

Protocol 2. Generation of *Hes7^BAP^* knock-in mice by homologous recombination

We inserted 42 bp (GGC CTG AAT **GAT ATC** TTT GAG GCC CAG AAG ATC GAG TGG CAT) encoding the 14-aminoacid BAP-tag (GLNDIFEAQKIEWH; Beckett et al 99) into the fourth exon of *Hes7* such that the tag is inserted 17 amino acids upstream of the Hes7 C-terminus. To this end, upstream and downstream fragments (1,379 bp and 5151 bp, respectively) were amplified by PCR using primers that incorporated the insert, and joined via the *EcoR*V site within the inserted tag (see sequence above, bold; for primer sequences see Table S1). The resulting 4,575 bp fragment was used as the 3’ homology region of the targeting construct. The adjacent upstream 1,042 bp fragment comprising of the second and third *Hes7* exon, the 2^nd^ and parts of the 1^st^ and 3^rd^ intron were PCR-amplified and served as 5’ homology region. The two homology regions flanked the floxed *neo^r^* gene.

Homologous recombinant mice were generated following standard protocols. The *neo^r^* gene was removed by crossing in *PGK*-driven Cre recombinase. The resulting *Hes7^BAP^* locus is identical to the wildtype *Hes7* locus with the exception of the inserted BAP tag and a remaining loxP site in the 3^rd^ intron. Heterozygous mice were intercrossed to remove the *PGK-cre* gene and to obtain homozygotes.

**Protocol 3. Whole mount in situ hybridisation of mouse embryos.**

Formaldehyde-fixed, proteinase K-treated embryos were pre-hybridised in hybridisation buffer (50% formamide; 1.3xSSC, pH5.0; 5 mM EDTA; 0.2% Tween-20; 0.5% CHAPS; 50 µg/ml yeast RNA; 100 µg/ml heparin) at 70°C for >1 h. Hybridisation with DIG-labelled RNA probes was performed overnight at 70°C. Hybridised embryos were washed in hybridisation buffer at 70°C for 1 h and in TBST (0.25 M Tris-HCl, pH7.5; 1.37 M NaCl; 27 mM KCl; 1% Tween-20) at room temperature for several hours, and subsequently incubated in TBST/10% heat-treated goat serum for >1 h and in alkaline-phosphatase coupled anti-DIG antibody (Roche; in TBST/10% goat serum) at 4°C over night. After extensive washes in TBST, embryos were transferred to NTMT (100 mM NaCl; 100 mM Tris-HCl, pH 9.5; 50 mM MgCl_2_; 10% Tween-20) where colour reaction was performed with NBT/BCIP (Roche) at room temperature for several hours.

**Reference.** Beckett, D., Kovaleva, E. and Schatz, P. J. (1999). A minimal peptide substrate in biotin holoenzyme synthetase-catalyzed biotinylation. *Protein Sci.* 8, 921-929.
